# No association between catechol-o-methyltransferase Val108/158Met polymorphism and schizophrenia or its clinical symptomatology in a Mexican population

**DOI:** 10.1007/s11033-012-2264-x

**Published:** 2012-11-27

**Authors:** Carlos Tovilla-Zárate, Beatriz Camarena Medellín, Ana Fresán, Lilia López-Narváez, Thelma Beatriz Gonzalez Castro, Isela Juárez Rojop, Julián Ramírez-Bello, Alma Genis, Humberto Nicolini

**Affiliations:** 1 División Académica Multidisciplinaria de Comalcalco, Universidad Juárez Autónoma de Tabasco, Ranchería Sur, Cuarta Sección, C.P. 86650 Comalcalco, Tabasco Mexico; 2 Depto. de Genética Psiquiátrica, Instituto Nacional de Psiquiatría Ramón de la Fuente Muñiz, Mexico City, D.F. Mexico; 3 CIGEN, Centro de Investigación Genómica, Comalcalco, Tabasco Mexico; 4 Laboratorio de Inmunogenómica y Enfermedades Metabólicas, Instituto Nacional de Medicina Genómica (INMEGEN), Mexico City, D.F. Mexico; 5 Grupo de Estudios Médicos Y Familiares Carracci, Mexico City, D.F. Mexico

**Keywords:** Schizophrenia, COMT, Association, Mexican population, Clinical symptomatology

## Abstract

The gene coding for catecol-o-methyltransferase (COMT), participant in the metabolism of catecholamines, has long been implicated as a candidate gene for schizophrenia. We determined the relation of the COMT Val108/158Met polymorphism with schizophrenia or its symptomatology (negative, disorganized and psychotic dimension). We conducted a case–control study comprising 186 patients with schizophrenia and 247 controls. The diagnosis of schizophrenia was established using the DSM-IV criteria for this illness. The clinical symptomatology was assessed through the Scale for the Assessment of Negative Symptoms and the Scale for the Assessment of Positive Symptoms. No significant differences were found in the distribution of alleles (χ2 = 0.01, df = 1, *p* = 0.90) or genotypes (χ2 = 1.66, df = 2, *p* = 0.43) between schizophrenic patients and the control group. Multivariate analysis showed that the COMT Val108/158Met polymorphism has no influence in the clinical symptomatology of schizophrenia. Our results showed no association between COMT Val108/158Met and schizophrenia or evidence for an association between COMT and the clinical symptomatology of this illness. This suggests that the COMT gene may not contribute to the risk for schizophrenia among the Mexican population.

## Introduction

Schizophrenia is a complex, debilitating psychiatric disorder characterized by positive and negative symptoms as well as cognitive disturbances. The median lifetime of this disorder is 4 per 1000 and its lifetime morbidity is 7.2 per 1,000 [1, 2]. Genetics plays a role in the etiology of schizophrenia with an estimated 0.81 inheritance approximately [3]. Several lines of evidence suggest that dysfunction in the dopaminergic system could lead to the development of schizophrenia and may account for the psychotic symptomatology of this illness [4, 5]. Given its position and function, the COMT gene has been proposed as a candidate gene involved in this pathology.

The gene coding for catecol-o-methyltransferase (COMT) is located on the long arm of chromosome 22 at 22q11; it spans 28 kb of the genome and contains six exons. A common polymorphism of the COMT gene is the Val108/158Met variant (rs4680). The low activity COMT genotype (COMTMet/Met), consisting of a Met/Met allele pair, yields a 3–4 fold lower enzyme activity compared to the high activity genotype (COMTVal/Val), which has a Val/Val allele pair, whereas the COMTVal/Met genotype produces intermediate enzyme activity [6]. However, the association between COMT and schizophrenia remains controversial, given that to date family-based association studies and case–control studies include positive and negative findings [7–15]. Moreover, evidence for COMT associations with behavioral features of schizophrenia has been reported [15–20]. Interestingly, most studies have found that the presence of the high activity Val allele, which is associated with lower levels of dopamine in the prefrontal cortex, predicts a poorer cognitive performance and outcome and the presence of brain abnormalities [21–23]. With regard to clinical phenotypes, some characteristics present in psychotic disorders have been studied such as age of onset, cognitive function, and symptomatology [17, 21, 24–26]. Therefore, to explore the possibility that COMT Val108/158Met polymorphism may have susceptibility for schizophrenia, we conducted a case–control study in a Mexican population. In addition, we evaluated the association between COMT Val108/158Met polymorphism and clinical symptomatology (age of onset and psychotic symptomatology of this disorder).

## Methods

### Subjects

A total of 186 schizophrenic patients were included in this study. Patients with schizophrenia were consecutively recruited from the outpatient services of the National Institute of Psychiatry Ramón de la Fuente and the Carracci Medical Group in Mexico City. All subjects signed an informed consent to participate in the study after they were given a verbal and written explanation of the research objectives. The study was approved by the ethics committee of the Carracci Medical Group and performed in accordance with the ethics standards laid down in the 1975 Declaration of Helsinki.

Diagnosis of schizophrenia was established by a trained psychiatrist using the Spanish version of the Diagnostic Interview for Genetic Studies (DIGS), a semi-structured interview which provides DSM-IV diagnosis as inclusion criteria. Exclusion criteria were: age younger than 15 years old or older than 60; current substance abuse, history of substance dependence, history of bipolar disorder, concomitant medical or neurological illness, and intellectual disability. To reduce ethnic variation and stratification effects, only Mexican subjects descending from Mexican parents and grandparents participated in this study.

COMT data were available from 186 subjects (108 males and 78 females; mean age: 30.8 years old; age range: 15–60 years; mean education level: 9.7 years, and education range: 1–19 years). The average age for the onset of illness was 21.3 ± 6.1 years old (males 20.33 ± 4.9, females 22.83 ± 7.3). The diagnosis of schizophrenia according to its spectrum was: paranoid 79.9 %, disorganized 13.2 % and catatonic 6.9 %. The control group included 222 unrelated subjects (116 males and 106 females). The mean age was 31.97 ± 12.84 years old. They were recruited from Blood Donor Center of the General Hospital of Comalcalco and from the general population of the Comalcalco city area in the state of Tabasco, México. Subjects were physically healthy on medical evaluation. All were of Mexican descent and none manifested psychiatric problems, as assessed in brief interviews by psychiatrists. Informed consent was obtained from each control subject.

### Assessments: clinical variables

Three clinical domains were proposed to establish the association between the COMT Val108/158Met genotype and clinical symptomatology of schizophrenia: negative, disorganized and psychotic symptomatology, together with the age of onset of the illness. Clinical symptomatology was assessed before pharmacological treatment.

### Psychotic, positive, and negative symptoms

The Scale for the Assessment of Negative Symptoms (SANS) [27] and the Scale for the Assessment of Positive Symptoms (SAPS) [28] were used to evaluate general psychopathology and symptom severity. The SANS scale comprises 25 items designed to assess five categories: (1) affective flattening, (2) alogia, (3) avolition/apathy, (4) anhedonia, and (5) attention. The SAPS scale consists of 30 items grouped in four categories: (1) hallucinations, (2) delusions, (3) bizarre behavior, and (4) positive formal thought disorder. Both scales have a scoring range from 0 to 5, where “0” denotes the absence of a particular symptom and “5” its most severe form. We divided the psychopathology into three dimensions: (a) negative, including all five categories of SANS, (b) disorganized, comprising the total scores of categories 3 and 4 of SAPS, and (c) psychotic, considering categories 1 and 2 of SAPS. These dimensions were calculated by following previous procedures reported in the literature [29, 30].

### Age of onset

Age of onset of the illness was defined as the age when patients experienced overt positive symptoms (hallucinations and delusions) for the first time; this parameter was obtained by a clinical interview with the patient and his/her relatives.

### COMT val108/158met (rs4680) genotyping

Genomic DNA was extracted from peripheral blood leukocytes using a modified version of the protocol by Lahiri [31]. The SNP rs4680 was genotyped as previously reported [32].

### Statistical analysis

Hardy–Weinberg equilibrium was tested using Pearson′s goodness-of-fit Chi squared test. The Chi squared test or Fisher′s Exact test was used to compare genotype and allele frequencies between cases and control group, and between COMT genotype and gender. Given the size of the sample, the power to detect associations was analyzed using the Quanto 1.2 software. The power of the analysis was 0.05. The level of significance was set at *p* < 0.05.

A Multivariate General Linear model (multivariate analysis of variance, MANOVA) was used to identify differences in the quantitative variables of interest (psychotic, disorganized and negative dimensions, as well as age of onset were used as dependent variables) among genotypes, thus avoiding the effects of multiple comparisons. COMT genotype and gender were used as factors to identify potential genotype–gender interactions and to control the effects of gender in COMT activity.

## Results

Genotype frequencies in the patient (*p* = 0.19) and control (*p* = 0.62) groups satisfied the Hardy–Weinberg equilibrium. The distribution of alleles and genotypes is presented in Table 1. No significant association was observed between cases and control group for genotype (χ2 = 1.66, df = 2, *p* = 0.43) or allele (χ2 = 0.01, df = 1, *p* = 0.90). When we analyzed the cases for gender, no differences were observed for genotype or allele (Table 1).

**Table 1:** Genotypes, alleles and frequency distributions of COMT polymorphisms in controls and patients with schizophrenia in a Mexican population

	Genotypic association *n* (%)			χ2	*p*	Allelic association *n* (%)		χ2	*p*
	Val/Val	Val/Met	Met/Met			Val	Met		
All									
Case	67 (36.0)	82 (44.0)	37 (20.0)	1.66	0.43	216 (58.0)	156 (42.0)	0.01	0.90
Control	72 (32.4)	112 (50.4)	38 (17.2)			256 (57.6)	188 (42.4)		
Male									
Case	37 (34.3)*	51 (47.2)	20 (18.5)	0.009	0.99	125 (57.8)	91 (58.3)	0.005	0.98
Control	36 (31.1)	62 (53.4)	18 (15.5)			134 (57.7)	98 (42.3)		
Female									
Case	30 (38.5)	31 (39.7)	17 (21.8)	1.00	0.60	91 (42.2)	65 (41.7)	0.13	0.71
Control	36 (33.9)	50 (47.2)	20 (18.9)			136 (60.1)	90 (39.9)		

In addition to COMT, the present study also focuses on the description of relevant socio-demographic and clinical characteristics of the patients distributed by genotypes and by negative, psychotic, or disorganized dimension (Table 2). No significant differences were observed in psychotic, negative, or disorganized dimension (Table 2). These results suggest that COMT Val108/158Met does not influence the severity of clinical symptoms in patients with schizophrenia.

**Table 2:** Demographic and clinical characteristics of patients with schizophrenia and distributions by COMT genotype

	Val/Val (*n* = 67)	Val/Met (*n* = 82)	Met/Met (*n* = 37)	Total sample	F	*p*
Education (mean ± SD)	10.04 ± 3.5	9.67 ± 3.7	9.65 ± 3.08	9.80 ± 3.50	0.25	0.77
Age (mean, SD)	31.12 (8.92)	30.43 (8.60)	31.41 (10.38)	30.81 (9.02)	0.17	0.83
Age of onset (mean, SD)	21.67 (5.89)	20.83 (6.52)	21.92 (6.04)	21.35 (6.19)	0.53	0.58
Psychotic dimension (mean, SD)	4.10 (3.31)	4.73 (3.17)	4.49 (3.04)	4.46 (3.19)	0.709	0.49
Disorganized dimension (mean, SD)	2.46 (2.09)	2.80 (2.16)	2.68 (2.12)	2.66 (2.12)	0.476	0.62
Negative dimension (mean, SD)	12.33 (5.42)	11.83 (5.30)	11.97 (5.27)	12.04 (5.3)	0.16	0.84

Results of multivariate ANOVA

*ANOVA* analysis of variance, *SD* standard deviation

The tests for the between-subjects effects in the MANOVA analysis for COMT, gender, and COMT-gender interaction are shown in Table 3. When analyzing the COMT genotype by gender interaction, no significant differences were encountered.

**Table 3:** Results of between-subjects effects using multivariate ANOVA

Factor	Dependent variable	F	*p*	Observed power
COMT	Age of onset	0.38	0.68	0.11
	Psychotic dimension	1.42	0.24	0.30
	Disorganized dimension	0.97	0.38	0.21
	Negative dimension	0.04	0.95	0.05
Gender	Age of onset	5.71	0.01	0.66
	Psychotic dimension	0.11	0.73	0.06
	Disorganized dimension	0.001	0.975	0.05
	Negative dimension	0.01	0.92	0.05
COMT x gender interaction	Age of onset	0.08	0.91	0.36
	Psychotic dimension	2.03	0.13	0.41
	Disorganized dimension	1.74	0.17	0.36
	Negative dimension	0.32	0.72	0.55

*COMT* catechol-o-methyltransferase

## Discussion

In this study, a population of Mexican schizophrenic patients was investigated to explore the association between COMT Val108/158Met and schizophrenia. Initially, a case–control study was conducted. Subsequently, we investigated whether the COMT Val108/158Met influences the severity of clinical symptoms. In our population, we found no association between the COMT polymorphism and schizophrenia by allele or genotype in the sample taken as a whole or by gender. Finally, no association between COMT Val108/158Met and severity of clinical symptoms was observed, but a significant diminution in the time of duration of untreated psychosis was observed in patients with the Met/Met genotype.

To our knowledge, this is the first study addressing the genetic association between COMT Val108/158Met and schizophrenia in a Mexican population. Our results are in agreement with case–control reports [13, 20, 33] and meta-analyses in the literature [34–36]. Similarly, a recent meta-analysis reported no association or protective effect of COMT Val108/158Met with schizophrenia [37]. However, some studies have shown positive association [38] or gender specific association [15] between COMT and schizophrenia. In our study this was not the case. A possible explanation for our results could be due to the stratification of the population. We observed differences in COMT genotype frequencies between our population and that of other studies. Our frequencies were similar to those encountered in a Caucasian population of Spain [29, 37] and in a Jewish Israeli population [25], but differed from a report in an Asian population [13, 20, 39]. These studies exemplify the heterogeneity in the genetic background among populations and corroborates the need of more studies for the replication of these results. Finally, the literature suggests that population stratification, sample size, and diagnostic criteria defining the sample could contribute to the lack of replication of the results in association studies [40].

Previous studies have suggested a relationship between the COMT Val108/158Met polymorphism and positive or negative symptoms in schizophrenia. [16, 18, 41]. This association is based on the notion that a decrease in dopamine activity in the dorsolateral prefrontal cortex contributes to negative symptoms and cognitive deficits. Our results do not agree with this hypothesis. However, they are in accordance with previous studies stating a lack of association between this polymorphism and clinical data [17, 20, 25, 26, 33]. One possibility for this outcome could be that another modifying gene is necessary so that the COMT polymorphism can manifest itself at the level of clinical expression in schizophrenia. If the allelic version of this hypothetical gene has a variable frequency in the population, there may be varying responses to the COMT polymorphism in schizophrenic patients from different ethnic groups. Similarly, the COMT polymorphism may have a differential influence in clinical symptomatology depending on the underlying genetic factors responsible for schizophrenia in each individual [17, 25].

In this study, the small number of patients limits the generalization of our findings. In addition, although the subjects were all of Mexican origin, we can not completely exclude the possibility of a population structure effect in our sample. Finally, we did not evaluate the antipsychotic medication in our patients who were being treated with a variety of drugs.

In conclusion, our results suggest that the functional Val108/158Met polymorphism of the COMT gene exhibited a lack of association between this polymorphism and schizophrenia in a sample of Mexican schizophrenic patients. Similarly, no association could be established with severity of SANS, SAPS-psychotic dimension, or SAPS-disorganized dimension in patients with this illness. However, further studies are required to gain more insight into the association of COMT polymorphism with either schizophrenia or its symptomatology.
